# Development and Assessment of a Social Media–Based Construct of Firearm Ownership: Computational Derivation and Benchmark Comparison

**DOI:** 10.2196/45187

**Published:** 2023-06-13

**Authors:** Carole Roan Gresenz, Lisa Singh, Yanchen Wang, Jaren Haber, Yaguang Liu

**Affiliations:** 1 Department of Health Management and Policy, McCourt School of Public Policy Georgetown University Washington, DC United States; 2 Department of Computer Science, Massive Data Institute Georgetown University Washington, DC United States; 3 Department of Computer Science Georgetown University Washington, DC United States; 4 Quantitative Social Science Dartmouth College Hanover, NH United States

**Keywords:** criterion validity, firearms ownership, gun violence, machine learning, social media data

## Abstract

**Background:**

Gun violence research is characterized by a dearth of data available for measuring key constructs. Social media data may offer a potential opportunity to significantly reduce that gap, but developing methods for deriving firearms-related constructs from social media data and understanding the measurement properties of such constructs are critical precursors to their broader use.

**Objective:**

This study aimed to develop a machine learning model of individual-level firearm ownership from social media data and assess the criterion validity of a state-level construct of ownership.

**Methods:**

We used survey responses to questions on firearm ownership linked with Twitter data to construct different machine learning models of firearm ownership. We externally validated these models using a set of firearm-related tweets hand-curated from the Twitter Streaming application programming interface and created state-level ownership estimates using a sample of users collected from the Twitter Decahose application programming interface. We assessed the criterion validity of state-level estimates by comparing their geographic variance to benchmark measures from the RAND State-Level Firearm Ownership Database.

**Results:**

We found that the logistic regression classifier for gun ownership performs the best with an accuracy of 0.7 and an *F*_1_-score of 0.69. We also found a strong positive correlation between Twitter-based estimates of gun ownership and benchmark ownership estimates. For states meeting a threshold requirement of a minimum of 100 labeled Twitter users, the Pearson and Spearman correlation coefficients are 0.63 (*P*<.001) and 0.64 (*P*<.001), respectively.

**Conclusions:**

Our success in developing a machine learning model of firearm ownership at the individual level with limited training data as well as a state-level construct that achieves a high level of criterion validity underscores the potential of social media data for advancing gun violence research. The ownership construct is an important precursor for understanding the representativeness of and variability in outcomes that have been the focus of social media analyses in gun violence research to date, such as attitudes, opinions, policy stances, sentiments, and perspectives on gun violence and gun policy. The high criterion validity we achieved for state-level gun ownership suggests that social media data may be a useful complement to traditional sources of information on gun ownership such as survey and administrative data, especially for identifying early signals of changes in geographic patterns of gun ownership, given the immediacy of the availability of social media data, their continuous generation, and their responsiveness. These results also lend support to the possibility that other computationally derived, social media–based constructs may be derivable, which could lend additional insight into firearm behaviors that are currently not well understood. More work is needed to develop other firearms-related constructs and to assess their measurement properties.

## Introduction

Social media data are a relatively new and potentially valuable source of information to support firearms research, an area long hindered by substantial data deficiencies [1-3]. Already, studies have used social media data in a variety of firearms-related research contexts, including (1) tracking sentiment, emotion or attitudes, and opinions around gun policy following mass shooting events [4-12]; (2) assessing the mental health–related effect of firearms violence and its sequelae [13,14]; (3) examining social media content related to firearm injury [15,16]; and (4) exploring the influence of firearms-related social media advertising [17,18]. Social media–based strategies to prevent gun violence represent a burgeoning area of additional research [19-21]. Although distinct from social media data, search data have also increasingly been used for firearms-related research questions, such as to examine gun purchasing and gun preparation behavior [22-24].

The strengths and limitations of social media data for advancing firearms research have been articulated from a conceptual perspective [1]. One strength, for example, is that social media data offer a unique capacity for capturing temporal dynamics, given the data’s continuous creation and near real-time availability. Previous research affirms the measurement property of responsiveness—defined as the ability of a measure to capture meaningful change [25]—for firearms-related social media–based constructs using a case study of firearms fatalities [26]. In contrast, social media data have limitations to generalizability distinct from survey or administrative data, where population representativeness is achieved by design (in the case of the former) or known by construct (in the case of the latter). These are just 2 dimensions of social media data among many that contribute to its promise or require special attention when these data are used for research [1].

Advancing the application of social media data to firearms research requires buttressing our conceptual understanding with methodological tools and empirical evidence to support the effective and appropriate use of these data. In particular, we are concerned with the derivation of firearm-related constructs from social media data and assessment of their measurement properties. Methodology research in this space is emerging [27,28], but there is only one example of the development of a Twitter-based firearm-related construct that we are aware of [26]. That work focused on firearm mortality, whereas this paper focuses on gun ownership.

More specifically, this study explores whether an accurate classifier of gun ownership at the individual level can be developed within a resource-constrained environment, where limited high-quality training data are available. We also examine the criterion validity—defined as the extent of agreement between the measure and a gold standard measure of the same concept [25]—of this construct at the state level. We do so by comparing the geographic variance in state-level estimates from our social media-derived construct to that from benchmark estimates from the RAND State Level Firearm Ownership Database [29]. The benchmark data represent state-of-the-art estimates of state-level gun ownership based on statistical models that blend information on ownership from 4 available survey data sources as well as proxies for ownership and background check measures [29]. Assessing the measurement properties of constructs derived from social media matters for determining the appropriateness of the use and interpretation of these constructs.

## Methods

### Overview

We constructed a machine learning model for firearm ownership and develop state-level firearm ownership estimates. Our primary data source is Twitter data from 2019 to 2021. We analyzed the correlation across states between these Twitter-derived estimates and benchmark ownership estimates from the RAND State-Level Firearm Ownership Database [29]. Although demographic information for Twitter users is generally limited, we have been able to build on previous machine learning research [30] to develop a reliable model for inferring gender for Twitter users. We used this to create gender-adjusted Twitter-based ownership estimates and in a sensitivity analysis to compare gender-adjusted Twitter-based estimates to benchmark ownership estimates.

### Ethics Approval

This research was approved by Georgetown University’s institutional review board (IRB; STUDY00002133). The consent procedures for the original survey panel recruitment informed the participants that invitations would be sent through email, surveys would be completed on the web, and the survey could be paused and completed later. Given that the research presented no more than minimal risk of harm to participants and involved no procedures for which written consent is normally required outside of the research context, a waiver of documentation of informed consent was provided (as per HHS.gov definition of waiver of signed consent). The Georgetown team did not provide compensation to survey participants as these data were collected by a different institution. The original data collection (approved by the University of Michigan IRB; HUM00151891) provided payment vouchers to participants of the study for up to US $25, depending on the frequency of their participation in the panel. Participants were given a US $5 incentive for providing their Twitter handle.

We conduct multiple predictions (gun ownership and gender) that have ethical implications. Although automated models provide valuable insight into people’s behaviors, errors can lead to equity issues. We also believe that privacy expectations should not be compromised. For this reason, we use data sets that either have an expectation of being public (Decahose and Gun Conversation Sample) or ones we obtain consent to use for research purposes (Linked Survey or Twitter Sample). We also choose to run all of our experiments on Twitter, where users do not typically have an expectation of privacy.

### Data

**Figure 1:**
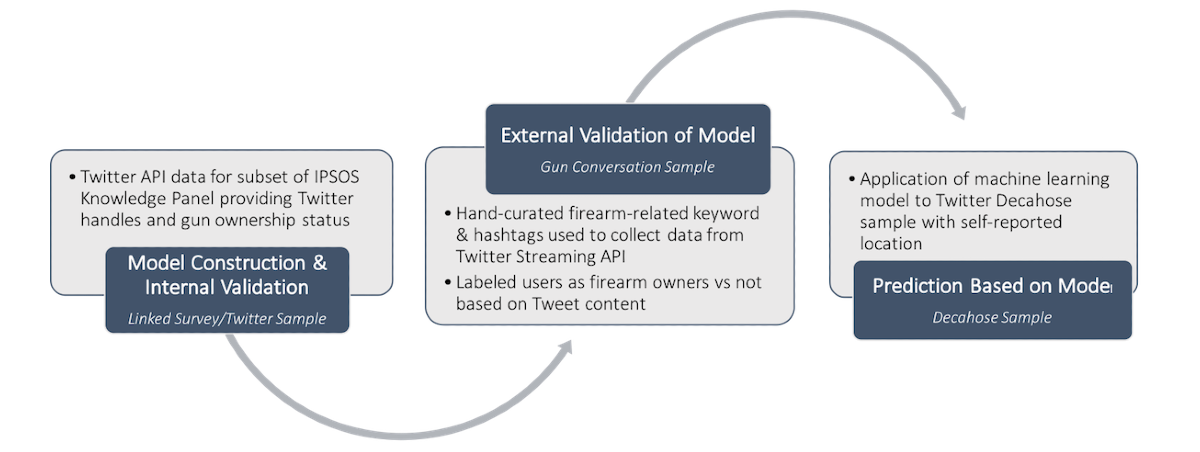
Three data sets used for machine learning model construction, validation, and estimation for gun ownership. API: application program interfaces.

We use Twitter application programming interfaces (APIs) to develop 4 data sets. Figure 1 summarizes 3 data sets we construct for the gun ownership machine learning process. The first data set is used to develop a machine learning model for determining gun ownership, the second for external validation of the machine learning model, and the third for computing, based on the validated machine learning model, firearm ownership estimates at the state level. We also created a fourth data set to construct and internally validate a machine learning model for determining gender.

We constructed the *Linked Survey* or *Twitter Sample* (Figure 1) to develop a machine learning model for determining gun ownership. The *Linked Survey* or *Twitter Sample* enables us to use an auxiliary source that links Twitter users with the outcome of interest (in our case, gun ownership). This data source is an exogenous source containing individual gun ownership information, as opposed to being “automatically derived” from the content of social media data itself. More specifically, a random sample of 2653 Ipsos KnowledgePanel respondents [31] from 2020 was asked whether they own a firearm and if they were Twitter users, whether they would consent to link their survey responses and their Twitter data for 12 months. A total of 677 respondents (25.5% of our Ipsos sample) who responded to the question of their firearm ownership provided their Twitter handles and consented to the data linkage. We create the *Linked Survey* or *Twitter Sample* by collecting and linking these users’ Twitter data to their survey responses, and we use these data to construct our machine learning model of firearm ownership. In general, 677 is a small number from which to construct our model, thereby making this task one that is constrained by the limited size of training data set.

The second data set we developed, the *Gun Conversation Sample* (Figure 1)*,* is for externally validating the machine learning model for gun ownership. We first hand-curated a set of ≥200 firearm-related keywords and hashtags (see Multimedia Appendix 1) by reviewing content from the Twitter search interface and using previous literature [32-34]. We query the Twitter Streaming API using this set of keywords and hashtags to identify a set of Twitter users engaged in English-language conversations about guns during 2021. We manually labeled a sample of these users as either owning a firearm or not owning a firearm by searching their tweet content for indicative phrases and hashtags (eg, “I am not a gun owner” or #iownguns). We selected users who have at least 20 tweets and use up to 300 of their most recent tweets for determining ownership. The majority of the tweets we collected are from 2021, but because people share tweets at different rates, some users’ most recent 300 tweets included those from 2020 and 2019. We successfully labeled 977 Twitter users as gun owners and 978 Twitter users as gun nonowners to create a final *Gun Conversation Sample* of labeled users for external validation.

Third, we developed a *Decahose Sample* (Figure 1) to calculate Twitter-based state firearm ownership estimates. We derived this data set from the Twitter Decahose API, which is a daily 10% random sample of all tweets. For state-level prediction purposes, we require information on Twitter users’ locations, but not all users provide their location as part of their biographical data, and it is not possible to sample the Decahose API from among those who do report their location. We instead sampled 10,000 users from the Decahose API who posted a geotagged tweet from a US location in English and checked these users’ biographical information for a self-reported, valid US location. This results in a final *Decahose Sample* of 7459 users. Although other studies have inferred location or used location mentions in posts, we chose to use a method that has high reliability.

We followed a similar process for constructing a machine learning model for determining gender and for internal or external validation of that model. To construct the machine learning model for gender, we collected tweets from the Twitter API for a publicly available research data set of 14,315 Twitter handles for which gender information is available [30]. We used this *Gender Sample* for constructing and internally validating the machine learning model, and we use the *Linked Survey/Twitter Sample* (which also includes gender information about respondents) for external validation.

Lastly, we use the RAND State-Level Firearm Ownership Database as our benchmark data [29]. This database provides estimates of state-level gun ownership based on statistical models that blend information on ownership from 4 available survey data sources as well as proxies for ownership and background check measures [29]. We used estimates from 2016, the most recent year for which estimates are available. Although our Twitter-based estimates are based on data primarily from 2021, geographic dispersion in gun ownership has exhibited relative stability [29].

### Machine Learning Model Construction

Our primary machine learning task is to build a model for predicting gun ownership. We used the *Linked Survey* or *Twitter Sample* for model construction using 5-fold cross-validation and holding out 10% of the sample for internal model validation. We explored the performance of a range of models using the sklearn Python package, including support vector machine, logistic regression, random forest, decision trees, and recurrent neural networks with and without an attention mechanism [35]. Our goal is to compare the performance of classic machine learning models and a fairly standard neural network model. We consider this neural model with and without an attention mechanism because attention mechanisms have performed well on other tasks using Twitter data [36,37]. For all classic (nonneural) models, we used N-gram features from the tweets and N-gram features from the profiles. We only considered original tweets and ignored retweets. We also only selected handles with more than 10 original tweets, having empirically determined that fewer than 10 original tweets are insufficient for reasonable prediction accuracy. For models that use N-gram features, we used the tweet tokenizer in the NLTK python package to tokenize each original tweet. During tokenization, we converted all text into lowercase and remove all stop words using stop words in the NLTK package. After tokenization, we used the N-gram Python package to convert tokens into unigram, bigram, and trigram. We then determined the frequency of unigram, bigram, and trigram in each tweet and we summed up all the frequency counts for each handle. For unigram, bigram, and trigram, we selected the top 400 most frequent tokens as our features. For each handle, we also constructed the following features from each profile: number of tweets, number of days since the first tweet, number of followers, number of followings, average number of words per tweet, average word length per tweet, proportion of emojis per tweet, proportion of hashtags per tweet, proportion of punctuation per tweet, proportion of emojis in biography, proportion of hashtags in biography, proportion of punctuation in biography, number of words in the biography, average word length in the biography proportion of emojis in the biography, proportion of hashtags in the biography, and proportion of punctuation in the biography.

For the neural models, the input features are language embeddings of tweets using bidirectional encoder representations from transformers [38]. More specifically, we used the most recent 200 original tweets (all the tweets if the user has less than 200). We then used the sentence transformer package in Python to convert each tweet into a vector and average the vectors for each handle. If the handle has a biography, we went through the same process. If the handle does not have a biography, we used a vector of zeros as the embedding feature. We input these features into a recurrent neural network with a parameter of batch_size=32, hidden_size=800, num_layers=500, learning_rate=0.0005 using the package “pytorch” and the function torch.nn.LSTM(). We added an attention layer on top of the long short-term memory networks (LSTM) layer. We use torch.bmm from pytorch to construct the attention layer. During the training process, we used the default dropout rate and early stopping to prevent overfitting.

Finally, we conducted external validation of our machine learning model using the *Gun Conversation Sample.*

We applied our machine learning model for gun ownership to tweets from users in the *Decahose Sample* to estimate the probability *p*(*g*) that each user is a gun owner. We then used *p*(*g*) to classify gun ownership status: *p*(*g*)>0.6 was classified as a gun owner, *p*(*g*)<0.4 was classified as not gun nonowner, and 0.6≥*p*(*g*)≥0.4 was classified as indeterminate. We labeled gun ownership (either gun owner or not a gun owner, excluding those who are indeterminate) for 5415 of the 7459 Twitter users in the *Decahose Sample*. For 17 states, our reliably labeled sample includes more than 100 users.

For states that do not contain at least 100 reliably labeled Twitter users, we used a data augmentation strategy to increase the sample. Specifically, we augmented the sample using a random sample of 61,059 Twitter users from the Twitter Streaming API engaged in gun-related conversation (based on the hashtags and keywords in Multimedia Appendix 1). We selected only those users (15,962) who have their location in their biographical data and applied the machine learning algorithm for gun ownership. We added 1231 Twitter users who are reliably labeled as a gun owner or a gun nonowner to those states with a small sample size (n<100) in our *Decahose Sample*, balancing to ensure the sample augmentation maintains the gender distribution in the nonaugmented sample since this distribution is available to us. The augmented *Decahose Sample* comprises 6646 Twitter users and includes 100 or more users for 34 states.

A second machine learning task involved building a model for gender. We used the *Gender Sample* for model construction, and we again used 5-fold cross-validation and held out 10% of our data for internal validation. For preprocessing, we removed handles having less than 10 tweets and lowercased all words. Although many classic machine learning and neural models have been developed for gender inference, recent research has shown the significantly stronger performance of neural models for this prediction task when using Twitter features [39]. Therefore, we used the state-of-the-art neural model for gender inference. The architecture of this classifier consists of a recurrent neural network with an attention layer, and the input features are language embeddings constructed using the contrastive language–image pretraining neural model. We input these features into the recurrent neural network. We used the bidirectional gated recurrent unit (GRU) from the Python pytorch package. The parameters of GRU were set to batch_size=32 and hidden_size=256. The learning_rate was set to 0.0001. We added an attention layer on top of the GRU layer. Weight, bias, and context vector were randomly initialized for the attention layers and then normalized with a mean value of 0 (SD 0.05). We used the Adam update rule to optimize our model, which is from torch.optim.Adam() function. They are jointly learned during training. We used early stopping to prevent overfitting. Finally, we used imblearn to create balanced training data sets during cross-validation. For external validation, we used the *Linked Survey/Twitter Sample*, which provides self-reported gender.

### Statistical Analyses

We calculated state-level estimates of firearm ownership using the percentage of Twitter users classified as gun owners in each state and compared the geographic dispersion of these estimates and benchmark estimates using Pearson and Spearman correlation coefficients. We refined our analyses by examining the correlation between Twitter-based and benchmark estimates of gun ownership for only those states in which we have labeled data (ie, location and gun ownership are reliably labeled) for a threshold number of Twitter users. We defined this threshold using alternative cutoffs of 60, 80, and 100 users. Additionally, we compared the Twitter-based estimate of gun ownership and the benchmark estimate based on the quartiles of their respective distributions to capture the relative performance of the Twitter-based estimate across states.

We also examined the correlation between gender-adjusted Twitter-based estimates and benchmark estimates. Although demographic differences between Twitter users and the resident population that are consistent across states will not affect the estimated correlation, state-variant demographic differences could affect the correlation. Although demographic information for Twitter users was generally limited, we were able to build on previous machine learning research to develop a reliable algorithm for inferring gender for Twitter users. We applied our machine learning model for gender to the augmented *Decahose Sample* and weighted the Twitter-based sample to match the state-level gender distribution.

## Results

Table 1 summarizes the performance of the best machine learning model (logistic regression) for gun ownership (see Multimedia Appendix 2 for the performance of all the models) and our gender model in terms of accuracy and *F*_1_-scores. Accuracy represents the percentage of predicted values that match known values for a given construct and the *F*_1_-score is the harmonic mean of sensitivity and specificity (precision and recall). For both gun ownership and gender, we achieved reasonable *F*_1_-scores for performance for the internal validation data (0.69 and 0.88 for gun ownership and gender, respectively), and on the external validation data (0.64 for gun ownership and 0.73 for gender).

**Table 1:** Performance of machine learning classifiers for predicting gun ownership and gender.

Measure and data set (purpose)		Best classifier: accuracy	Best classifier: *F*_1_-score
**Gun ownership**			
	Linked survey or Twitter sample (cross-validation average)	0.72	0.69
	Holdout from linked survey or Twitter sample (internal validation)	0.71	0.70
	Gun conversation sample (external validation)	0.65	0.64
**Gender**			
	Gender sample (training)	0.90	0.87
	Holdout from gender sample (internal validation)	0.90	0.88
	Linked survey or Twitter sample (external validation)	0.74	0.73

Table 2 summarizes the results of our correlation analysis for gun ownership estimates for all states and for a subset of states that met our threshold requirement for the number of labeled Twitter users. For all states, the unadjusted correlations are weakly positive (*r*=0.36, =0.44) and statistically significant (*P*=.01 and *P=*.001). We refined our analyses by using only those states with at least 60, 80, or 100 Twitter users. We found that when the cutoff was 60, the correlations were lower by 0.1-0.15 than were those when using 80 or 100 as the cutoff. Although the correlations are similar when using a cutoff of 80 and 100, there was greater variability for states with less than 100 Twitter users; therefore, we used 100 as the cutoff for our sample in this part of the analysis. Table 2 reports the results for analyses restricted to the 34 states with 100 or more Twitter users. For these analyses, the correlations are strongly positive and statistically significant (*r*=0.63, *P*<.001; =0.64, *P*<.001).

**Figure 2:**
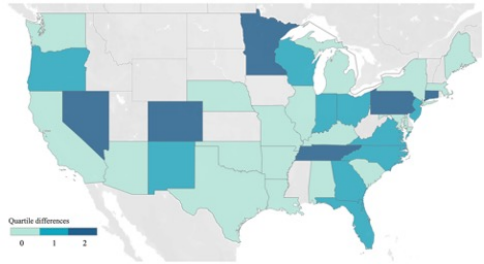
Comparison of Twitter-based estimates and benchmark estimates of gun ownership.

We provide a comparison of the Twitter-based estimate of gun ownership and the benchmark estimate based on quartiles of their respective distributions for states with at least 100 Twitter users in the sample (Figure 2). In half of the states, the Twitter-based estimate and the benchmark estimate fall into the same quartile of their respective distributions (represented in light blue in Figure 2). Furthermore, 35% of states have a single-quartile difference (shown in turquoise) and a 2-quartile difference exists for the remaining 15% of states (highlighted in dark blue). The cross-state differences in accuracy appear to be largely driven by sample size, as states with the largest quartile differences have an average of 113 users versus 162 and 189 users in states with 1 or no quartile differences, respectively.

**Table 2:** Correlation between Twitter-based and benchmark estimates of gun ownership.

Sample and subset		Pearson correlation			Spearman rank correlation	
		*r*	*P* value	ρ		*P* value
**Unadjusted**						
	All states	0.358	.01	0.438		.001
	States with ≥100 Twitter users	0.632	<.001	0.639		<.001
**Gender-adjusted**						
	All states	0.355	.01	0.427		.002
	States with ≥100 Twitter users	0.624	<.001	0.633		<.001

Finally, we show results for analyses with gender-adjusted Twitter-based estimates of gun ownership (Table 2). We found that gender adjustment has little impact on the estimated correlation, in terms of either magnitude or statistical significance.

## Discussion

### Principal Results

We developed a set of machine learning models for predicting gun ownership with limited training data and found that the logistic regression classifier for gun ownership performs the best with an accuracy of 0.7 and an *F*_1_-score of 0.69. This is considered reasonable classification performance when data are noisy and the size of the training data is small. To further improve the performance, significantly more data would need to be labeled, and it is unclear how much more data would be needed to get a significant improvement. Another direction would be to consider pretraining neural models with relevant Twitter data to provide a language model like bidirectional encoder representations from transformers or contrastive language–image pretraining with more domain-specific background knowledge.

Given an adequate sample of Twitter users in each state, which we defined as 100 handles, we found a strongly positive geographic (cross-state) correlation between Twitter-based gun ownership estimates and benchmark estimates. Our correlation results comparing the geographic dispersion of state-level estimates of ownership in our social media-based measure to that from a benchmark were, unsurprisingly, weaker when we included states for which our Twitter-based estimates rely on a small number (fewer than 100 users) of labeled Twitter users. This analytic challenge—the small number of labeled Twitter users despite large initial social media data draws coupled with data augmentation—underscores the importance of leveraging even larger draws of social media data in future research using location identification. The sample size issue also foregrounds the need to consider location imputation, including applying machine learning to nonbiographical data, such as tweet content and user follower or following networks [40].

We tested whether gender adjustment would better align the geographic dispersion in Twitter-based estimates with that of benchmark ownership estimates, and we found that found that the adjustment made little difference. We caution that this finding should not be interpreted as general evidence that analytic adjustments for gender or other demographic characteristics are empirically unimportant.

### Limitations

Limitations of our study include that our predictive data are restricted to individuals for whom a validated location (state) could be identified and for whom gun ownership could be reliably inferred, which resulted in small sample sizes in some states. Additionally, because social media data contain little individual-level demographic information about users, we were not able to analyze estimates adjusted for demographic attributes other than gender. Lastly, we are unable to account for potential changes in the geographic dispersion of gun ownership between the period of our benchmark estimates (2016) and our Twitter-based estimates (primarily from 2019).

### Conclusions

This study advances the application of social media data to gun violence research in several ways. First, we demonstrate the feasibility of creating a model using publicly available social media data for a key firearms-related construct—gun ownership. To date, a predominant focus in many firearms studies that use social media has been on attitudes, opinions, policy stances, sentiments, and perspectives related to mass shootings, other firearms events, and gun policy [1]. But in many cases, key characteristics of individuals who are posting to social media on firearms-related topics are unknown, including sociodemographic characteristics as well important context-specific characteristics, such as gun ownership. Linking characteristics of social media users with an analysis of social media content provides information about representativeness and allows analysts to explore variability in outcomes—sentiments, public opinion, health-related behaviors, or otherwise—across important user characteristics. This underscores the importance of continued work to develop reliable machine learning models for imputing a range of demographic and context-specific characteristics of social media users [41]. As more demographic inference models emerge, we need to have standard cross-disciplinary ways to assess their reliability on different data sets from across different social media platforms. For example, we chose a very reliable approach for determining location. However, other methods exist that could potentially lead to higher levels of coverage [42-44]. Future work should investigate the trade-off between reliability and coverage when inferring different demographics.

Additionally, we established the criterion validity of state-level ownership estimates based on the social media-based ownership construct. Assessing the measurement properties of constructs derived from social media matters for determining the appropriateness of the use and interpretation of these constructs. The high criterion validity we achieve for state-level gun ownership suggests that social media data may be a useful complement to traditional sources of information on gun ownership, such as survey and administrative data, and—given the immediacy of their availability, continuous generation, and responsiveness [1]—especially for identifying early signals of changes in geographic patterns of gun ownership. More work is needed to determine the extent to which social media data can provide information about geographic patterns in dimensions of ownership other than simply the extensive margin (ownership vs not), such as the number and type of firearms owned and characteristics of their storage or acquisition.

Our analytic approach to constructing data for the machine learning model of firearm ownership and our approach to demographic adjustment for representativeness provide examples of how these techniques can be used, and potentially expanded upon, in future gun violence research. These results lend support to the possibility that other computationally derived, social media–based constructs may be derivable, which could lend additional insight into firearm behaviors that are currently not well understood. More work is needed to develop other firearms-related constructs and to assess their measurement properties. As the frontiers of social media data promise for firearms research are explored, careful attention to analytical challenges associated with using these data, as well as to ethical standards for use, is essential.

## Appendix

Multimedia Appendix 1 Hashtags, keywords and phrases used to collect data from Twitter API.

Multimedia Appendix 2 Performance of Gun Ownership Machine Learning Labels.

## Abbreviations

API: application programming interface
GRU: gated recurrent unit
IRB: institutional review board
LSTM: long short-term memory

## Data Availability

The data sets generated and analyzed during this study are not publicly available because public sharing of the data would violate the terms of our data use agreement. However, the Massive Data Institute at Georgetown University houses a Gun Violence Text Analytic Portal that can be accessed by researchers to construct variables from Twitter conversations about guns. The portal is available at the Massive Data Institute’s website [45].
