# Supplementary material for: Development and Assessment of a Social Media–Based Construct of Firearm Ownership: Computational Derivation and Benchmark Comparison
Source: J Med Internet Res. 2023 Jun 13;25:e45187. doi: 10.2196/45187 (PMC10365610; doi:10.2196/45187)
Supplement: Multimedia Appendix 2 [file jmir_v25i1e45187_app2.pdf]

Multimedia Appendix 2: Performance of Gun Ownership Machine Learning Labels

Table S1: Performance of all machine learning models predicting gun ownership using linked survey/Twitter sample (cross-validation average)

Model	Accuracy	F1 score
**Logistic regression**	0.72	0.70
**Random forest**	0.64	0.50
**Decision tree**	0.60	0.48
**SVM**	0.65	0.56
**NN without attention layer**	0.65	0.59
**NN with attention layer**	0.69	0.62

Table S2: Performance of all machine learning models predicting gun ownership using holdout from linked survey/Twitter sample (internal validation)

Model	Accuracy	F1 score
**Logistic regression**	0.71	0.70
**Random forest**	0.66	0.52
**Decision tree**	0.62	0.50
**SVM**	0.64	0.57
**NN without attention layer**	0.66	0.60
**NN with attention layer**	0.69	0.63
